# Comorbidities Are Associated With Unfavorable Outcome in Aquaporin‐4 Antibody Positive Neuromyelitis Optica Spectrum Disorders and Myelin Oligodendrocyte Glycoprotein Antibody‐Associated Disease: Exploratory Study From the CROCTINO Cohort

**DOI:** 10.1111/ene.70214

**Published:** 2025-06-09

**Authors:** Sara Samadzadeh, Frederike Cosima Oertel, Hadi Salih, Ting‐Yi Lin, Seyedamirhosein Motamedi, Claudia Chien, Lawrence J. Cook, Marco Aurélio Lana‐Peixoto, Mariana Andrade Fontenelle, Ho Jin Kim, Jae‐Won Hyun, Su‐Kyung Jung, Jaqueline Palace, Adriana Roca‐Fernandez, Maria Isabel Leite, Srilakshmi M. Sharma, Fereshteh Ashtari, Rahele Kafieh, Alireza Dehghani, Mohsen Pourazizi, Lekha Pandit, Anitha Dcunha, Orhan Aktas, Marius Ringelstein, Philipp Albrecht, Eugene F. May, Caryl Tongco, Letizia Leocani, Marco Pisa, Marta Radaelli, Bernardo Sánchez‐Dalmau, Elena H. Martinez‐Lapiscina, Hadas Stiebel‐Kalish, Mark A. Hellmann, Itay Lotan, Sasitorn Siritho, Jérôme de Seze, Thomas Senger, Joachim Havla, Romain Marignier, Caroline Froment Tilikete, Alvaro Cobo‐Calvo, Denis Bichuetti, Ivan Maynart Tavares, Kerstin Soelberg, Ayse Altintas, Rengin Yildirim, Uygur Tanriverdi, Anu Jacob, Saif Huda, Zoe Rimler, Allyson Reid, Yang Mao‐Draayer, Pablo Villoslada, Ibis Soto de Castillo, Ari Green, Axel Petzold, Michael R. Yeaman, Terry J. Smith, Alexander U. Brandt, Hanna G. Zimmermann, Friedemann Paul, Nasrin Asgari, Guthy Jackson Charitable Foundation – International Clinical Consortium and CROCTINO Collaborators

**Affiliations:** ^1^ Institute of Regional Health Research University of Southern Denmark Odense Denmark; ^2^ The Center for Neurological Research Department of Neurology Slagelse Hospitals Slagelse Denmark; ^3^ Experimental and Clinical Research Center Max Delbrück Center for Molecular Medicine and Charité – Universitätsmedizin Berlin, Corporate Member of Freie Universität Berlin and Humboldt‐Universität zu Berlin Berlin Germany; ^4^ Max Delbrück Center for Molecular Medicine in the Helmholtz Association (MDC) Berlin Germany; ^5^ Neuroscience Clinical Research Center Charité – Universitätsmedizin Berlin, Corporate Member of Freie Universität Berlin and Humboldt‐Universität zu Berlin Berlin Germany; ^6^ Department of Neurology Johns Hopkins Hospital Baltimore Maryland USA; ^7^ Department of Psychiatry and Neurosciences Charité ‐ Universitätsmedizin Berlin Berlin Germany; ^8^ Department of Pediatrics University of Utah Salt Lake City Utah USA; ^9^ CIEM MS Research Center Federal University of Minas Gerais Belo Horizonte Brazil; ^10^ CIEM MS Research Center Medical School, University of Minas Gerais Belo Horizonte Brazil; ^11^ National Cancer Center Korea Goyang‐si South Korea; ^12^ Department of Neurology Oxford University Hospitals, National Health Service Trust Oxford UK; ^13^ Department of Ophthalmology Oxford University Hospitals, National Health Service Trust Oxford UK; ^14^ Kashani MS Center, Isfahan Neuroscience Research Center Isfahan University of Medical Sciences Isfahan Iran; ^15^ Department of Engineering Durham University Durham UK; ^16^ Department of Ophthalmology, Isfahan Eye Research Center Isfahan University of Medical Sciences Isfahan Iran; ^17^ Center for Advanced Neurological Research, KS Hegde Medical Academy Nitte University Mangalore India; ^18^ Department of Neurology, Medical Faculty Heinrich Heine University Düsseldorf Düsseldorf Germany; ^19^ Department of Neurology, Center for Neurology and Neuropsychiatry, LVR‐Klinikum Heinrich‐Heine‐University Düsseldorf Düsseldorf Germany; ^20^ Department of Neurology Kliniken Maria Hilf GmbH Mönchengladbach Mönchengladbach Germany; ^21^ Department of Medicine Los Angeles Biomedical Research Institute at Harbor‐University of California at Los Angeles (UCLA) Medical Center Torrance California USA; ^22^ Department of Medicine David Geffen School of Medicine at UCLA Los Angeles California USA; ^23^ University Vita‐Salute San Raffaele Milan Italy; ^24^ IRCSS Scientific Institute San Raffaele Milan Italy; ^25^ Department of Neurorehabilitation Sciences Casa di Cura Igea Milan Italy; ^26^ Neurological Department and Institute of Experimental Neurology (INSPE) Scientific Institute Hospital San Raffaele, University Vita‐Salute San Raffaele Milan Italy; ^27^ Department of Nuroscience Papa Giovanni Paolo XXIII Hospital Bergamo Italy; ^28^ Ophthalmology Department Hospital Clínic de Barcelona Universitat de Barcelona, IDIBAPS Barcelona Spain; ^29^ Hospital Clinic of Barcelona‐Institut d'Investigacions, Biomèdiques August Pi Sunyer (IDIBAPS) Barcelona Spain; ^30^ Neuro‐Ophthalmology Division, Department of Ophthalmology Rabin Medical Center Petah Tikva Israel; ^31^ Eye Laboratory, Felsenstein Medical Research Center Tel Aviv University Tel Aviv Israel; ^32^ Faculty of Medicine Tel Aviv University Tel Aviv Israel; ^33^ Department of Neurology, Rabin Medical Center, and Faculty of Medicine Tel Aviv Univeristy Tel Aviv Israel; ^34^ Siriraj Neuroimmunology Center, Division of Neurology, Department of Medicine Siriraj Hospital and Bumrungrad International Hospital Bangkok Thailand; ^35^ Neurology Service University Hospital of Strasbourg Strasbourg France; ^36^ Institute of Clinical Neuroimmunology, LMU Hospital Ludwig‐Maximilians‐University Munich Munich Germany; ^37^ Neurology, Multiple Sclerosis, Myelin Disorders and Neuroinflammation Pierre Wertheimer Neurological Hospital, Hospices Civils de Lyon Lyon France; ^38^ Department of Neuro‐Ophthalmology Hospices Civils de Lyon Lyon France; ^39^ Neurology Department, Centre d'Esclerosi Múltiple de Catalunya (Cemcat) Hospital Universitari Vall d'Hebron, Vall d'Hebron Institut de Recerca Barcelona Spain; ^40^ Departamento de Neurologia e Neurocirurgia, Escola Paulista de Medicina Universidade Federal de São Paulo São Paulo Brazil; ^41^ Department of Ophthalmology and Visual Sciences, Escola Paulista de Medicina Universidade Federal de São Paulo São Paulo Brazil; ^42^ Department of Clinical Immunology Odense University Hospital Odense Denmark; ^43^ Neurology Department, School of Medicine Koc University Istanbul Turkey; ^44^ Neurology Department Istanbul, Cerrahpasa School of Medicine Istanbul University Istanbul Turkey; ^45^ Department of Ophthalmology, Cerrahpasa Medical Faculty Istanbul University Fatih Turkey; ^46^ The Walton Centre for Neurology and Neurosurgery NHS Foundation Trust Liverpool UK; ^47^ Cleveland Clinic Abu Dhabi Abu Dhabi UAE; ^48^ Department of Neurology, NYU Multiple Sclerosis Comprehensive Care Center NYU School of Medicine New York New York USA; ^49^ Department of Neurology University of Michigan Medical School Ann Arbor Michigan USA; ^50^ Department of Neurology Hospital del Mar – Pompeu Fabra University Barcelona Spain; ^51^ Department of Neurology Hospital Clínico de Maracaibo Maracaibo Venezuela; ^52^ Department of Neurology University of California, San Francisco San Francisco California USA; ^53^ Faculty of Brain Sciences, UCL Queen Square Institute of Neurology University College London London UK; ^54^ The National Hospital for Neurology and Neurosurgery and Moorfields Eye Hospital London UK; ^55^ Department of Neurology Amsterdam UMC Amsterdam the Netherlands; ^56^ Department of Ophthalmology Amsterdam UMC Amsterdam the Netherlands; ^57^ Divisions of Molecular Medicine & Infectious Diseases, Department of Medicine Harbor‐UCLA Medical Center Torrance California USA; ^58^ Department of Ophthalmology and Visual Sciences Kellogg Eye Center Ann Arbor Michigan USA; ^59^ Division of Metabolism, Endocrine and Diabetes, Department of Internal Medicine University of Michigan Medical School Ann Arbor Michigan USA; ^60^ Department of Neurology Charité – Universitätsmedizin Berlin, Corporate Member of Freie Universität Berlin, Humboldt‐Universität zu Berlin, and Berlin Institute of Health Berlin Germany

**Keywords:** anti‐aquaporin‐4 (AQP4), anti‐myelin oligodendrocyte glycoprotein antibody‐associated disease, comorbidity, double‐seronegative NMOSD, neuromyelitis optica spectrum disorder, optical coherence tomography

## Abstract

**Background:**

Comorbidities occur in aquaporin‐4 antibody‐positive neuromyelitis optica spectrum disorder (AQP4‐NMOSD), myelin oligodendrocyte glycoprotein antibody‐associated disease (MOGAD), and double seronegative NMOSD (DN‐NMOSD), potentially contributing to a less favorable disease course.

**Objectives:**

To characterize comorbidities in AQP4‐NMOSD, MOGAD, and DN‐NMOSD and assess their association with optic neuritis (ON) outcomes by optical coherence tomography (OCT) in AQP4‐NMOSD.

**Methods:**

Four hundred and forty‐two participants from the CROCTINO cohort were evaluated for comorbidities.

**Results:**

In AQP4‐NMOSD patients (*n* = 360), 43.5% (*n* = 161) had comorbidities, equally divided between single and multiple. In MOGAD (*n* = 49), 40.8% had comorbidities, with 75% (*n* = 15) single and 25% (*n* = 5) multiple. In DN‐NMOSD (*n* = 33), 36.4% (*n* = 12) had comorbidities equally split. AQP4‐NMOSD patients had more multiple comorbidities (50%, *n* = 81/161) than MOGAD (25%, *n* = 5/20, *p* = 0.03) and more autoimmune disorders (AID) (40.4%, *n* = 65) than MOGAD (20%, *n* = 4, *p* = 0.09) and DN‐NMOSD (none, *p* = 0.004). Cardiovascular comorbidities and related risk factors (CVC/RF) occurred in 34.8% (*n* = 56) of AQP4‐NMOSD, 50% (*n* = 10) of MOGAD, and 33.3% (*n* = 4) of DN‐NMOSD. Expanded Disability Status Scale was higher in MOGAD (3.0 vs. 2.0, *p* = 0.006) and DN‐NMOSD (5.0 vs. 2.0, *p* = 0.008) with comorbidities. AQP4‐NMOSD patients with CVC/RF had higher ON relapse rates than those with AID (1.06 ± 3.33 vs. 0.49 ± 0.98, *p* < 0.001). OCT revealed reduced inner nuclear layer thickness in AQP4‐NMOSD with comorbidities compared to non‐comorbidity (*B* = −1.52, *p* = 0.047), more pronounced with CVC/RF (*B* = −2.96, *p* = 0.009).

**Conclusion:**

Comorbidities are frequent in AQP4‐NMOSD and MOGAD and are associated with ON frequency and disability. These findings highlight the need for proactive comorbidity management to improve patient care.

## 1 Introduction

Neuromyelitis optica spectrum disorder (NMOSD) is a rare inflammatory autoimmune disease of the central nervous system (CNS), characterized by damage to astrocytes with subsequent inflammation, demyelination, and neurodegeneration [1, 2, 3]. Serum immunoglobulin G autoantibodies (IgG) are found in the majority of NMOSD patients, targeting the astrocyte water channel aquaporin‐4 (AQP4) [4, 5, 6, 7]. Myelin oligodendrocyte glycoprotein (MOG) antibody‐associated disease (MOGAD) is a recently described entity with important differential diagnostic criteria compared to NMOSD. In MOGAD, antibodies (MOG‐IgG) target MOG, primarily associated with demyelination [8, 9, 10]. Individuals within the NMOSD spectrum who are seronegative for both AQP4‐IgG and MOG‐IgG antibodies are known as double seronegative (DN) NMOSD (DN‐NMOSD) and represent a heterogeneous subgroup [11, 12]. In all three clinical entities, optic neuritis (ON) is a frequent manifestation often associated with severe neurodegeneration and blindness [13].

ON is an inflammatory demyelinating condition characterized by primary inflammation, demyelination, and axonal injury in the optic nerve, which may lead to poor visual function [12, 14, 15, 16]. Optical coherence tomography (OCT) is a neuroimaging diagnostic tool that can be useful to differentiate AQP4‐NMOSD and MOGAD from distinct conditions such as multiple sclerosis (MS) [17, 18]. OCT can quantify neuro‐axonal retinal damage by measuring specific inner retinal layers, including the peripapillary retinal nerve fiber layer (pRNFL), the ganglion cell and inner plexiform layer (GCIPL), and the inner nuclear layer (INL) [19, 20, 21, 22, 23, 24, 25].

Emerging evidence suggests that comorbidities occur frequently, in particular in NMOSD, and may contribute to worsened clinical outcomes [22, 23, 24, 25, 26, 27, 28, 29]. Comorbidities refer to any additional condition co‐existing with the primary index disease in the same individual. Comorbidities may result from coincident predisposing genetic or environmental factors, immunologic mechanisms (e.g., antigen spreading) or from treatment of an index disease [30]. In MS, comorbidities have become an area of increasing interest in recent years [31] because they can adversely affect a broad range of outcomes, including the risk of relapse and disease progression, associated with diminished quality of life (QoL) and long‐term disability [22, 24, 25, 28, 29].

Exploring the epidemiology and clinical consequences of comorbidities in a large multicenter cohort may enhance generalizability and facilitate assessments comparing comorbidity patterns across AQP4‐NMOSD, MOGAD, and DN‐NMOSD. This may provide more information on comorbidity impact on clinical outcomes and may explain the heterogeneity in clinical results. We assessed comorbidities in a large cohort, the CROCTINO study (The Collaborative Retrospective Study of Retinal Optical Coherence Tomography in Neuromyelitis optica) with the associated OCT dataset. Visual outcomes and OCT were included because these were the outcome measures available in the CROCTINO Cohort and aligned with the aims of this study. This provided a valuable opportunity to analyze ON and investigate whether comorbidities influence ON outcomes with a level of detail not previously reported.

Building on this framework, in the current study, we investigated whether the frequency and type of comorbidities (a) differ among patients with AQP4‐NMOSD, MOGAD, and DN‐NMOSD; (b) interact with clinical outcomes; (c) impact retinal integrity after ON as measured by OCT in individuals with AQP4‐NMOSD.

## 2 Materials and Methods

### 2.1 Study Design and Participants

The Collaborative Retrospective Study of Retinal Optical Coherence Tomography in Neuromyelitis optica (CROCTINO) aimed to analyze retinal pathology using OCT in AQP4‐NMOSD (369), MOGAD (54) and DN‐NMOSD (58), with a total of 515 participants, who fulfilled the 2006 [32] and 2015 diagnostic criteria for NMOSD [33]. Data are reported according to STROBE reporting guidelines [34]. These data were collected from 22 participating centers located across North and South America, Asia, and Europe. Participating centers contributed OCT data and clinical metadata (acquired between 2000 and 2018). A detailed explanation of the dataset can be found elsewhere [12, 34]. Of note, the original design of the CROCTINO dataset was not intended to evaluate comorbidities. From the total CROCTINO cohort, 73 (14%) patients were excluded due to missing data on comorbidities, OCT results, or unknown antibody testing status. This exclusion left 442 patients eligible for this comorbidity study. Demographic and clinical data such as Expanded Disability Status Scale (EDSS), age at onset, time since onset, total number of ON attacks per person, and visual acuity were collected from all patients, along with OCT measurements.

### 2.2 Standard Protocol Approvals, Registrations, and Patient Consents

All participants gave written informed consent, and the study was approved by local ethics committees and conducted in accordance with the applicable laws and the current version of the Declaration of Helsinki.

### 2.3 Classification of Comorbidities

In the CROCTINO study, participating centers documented comorbidities in a free‐text field. The onset times of these comorbidities were not recorded. For the current analysis, we standardized and reclassified the data from the free‐text fields. Any records not originally in English were translated into English. All free‐text entries were carefully read and transformed into standard terminology, ensuring uniformity in the representation of the various conditions. The available data on comorbidity were then methodically classified into 14 principal categories. Categories included Cardiovascular and related risk factors (CVC/RF), autoimmune comorbidities (AID), endocrine, psychiatric, neurological, respiratory, gastrointestinal, neoplastic, rheumatologic, hematologic, dermatologic, genitourinary, infectious, and ophthalmic diseases. An additional category was created to incorporate any conditions not covered by the previous list. In this framework, injuries, and acute presentations, especially traumas and their associated fractures, were situated within the ‘injuries’ domain, rather than being identified as chronic comorbid conditions. If nicotine abuse was documented for a patient, it was included in the category “Cardiovascular and Related Risk Factors.” However, smoking was not systematically assessed. Of note, no included patient had ON‐unrelated ophthalmic comorbidities that could potentially interfere with OCT results (e.g., glaucoma). The details of the comorbidities included in all categories, particularly the CVC/RF and AID groups, are provided in Table S1.

### 2.4 Optical Coherence Tomography

Various OCT devices were used at each center. Only data from Spectralis SD‐OCT (Heidelberg Engineering, Heidelberg, Germany) were used in this study. Image quality was assessed using modified OSCAR‐IB criteria by experienced graders [35, 36, 37]. Eyes were excluded if neither the ring nor macular scan passed quality control. OCT measurements in the acute stage, within 3 months, were excluded in this current study. The peripapillary retinal nerve fiber layer (pRNFL) thickness was measured and corrected per protocol (peripapillary ring scan with a 12°‐ or 3.5‐mm diameter around the optic disc.) GCIPL and IPL were extracted from macular volume scans with a custom segmentation pipeline, as described earlier [34, 38]. The GCIPL thickness for the combined ganglion cell and inner plexiform layers was calculated based on the summation of both layers.

### 2.5 Statistical Methods

All statistical analyses were performed using R statistical software (version 4.3.1). Data were analyzed visually and statistically for their distribution. Next, descriptive statistics were performed based on the data distribution using the ‘base’ package in R to compute means, median, interquartile range, standard deviations, and frequencies. The distribution of comorbidities across different patient groups was examined using the “dplyr” and “tidyr” packages for data manipulation and summarization.

Welch Two Sample t‐test, one‐way ANOVA, and chi‐squared tests were conducted using the “stats” package. Logistic regression analyses were executed with the ‘survival’ package to identify age thresholds for comorbidity presence, and receiver operating characteristic (ROC) curve analysis was conducted using the ‘pROC’ package, which computed the area under the curve (AUC). A *p*‐value of less than 0.05 was considered significant. The study was exploratory with no sample size calculation and no adjustments were made for multiple comparisons.

Linear mixed‐effects models were conducted using the “nlme” package to assess the impact of comorbidity on OCT metrics. Two models were designed: the first, a simple model, was applied separately to two subsets of eyes those with ON and those without. This analysis included random effects for inter‐eye within‐subject variations and fixed effects for comorbidity, AID, and CVC/RF separately. The second, more complex model included random effects for inter‐eye within‐subject variations and fixed effects as above for ON, comorbidity or AID or CVC/RF status, and the interaction between ON and these parameters. The evaluated OCT metrics included pRNFL, GCIPL, and INL thicknesses.

## 3 Results

### 3.1 Comorbidity Distribution

**FIGURE 1:**
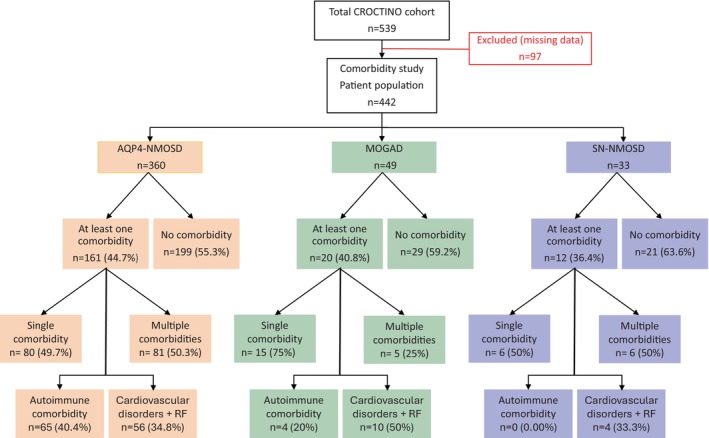
Distribution of comorbidity, cardiovascular comorbidities and risk factors, and autoimmune comorbidities among AQP4‐IgG seropositive neuromyelitis optica spectrum disorder (AQP4‐NMOSD), myelin oligodendrocyte glycoprotein antibody disease (MOGAD), and double seronegative with NMOSD phenotype (DN‐NMOSD). RF: risk factors.

The study included 442 patients, categorized into AQP4‐NMOSD (*n* = 360), MOGAD (*n* = 49), and DN‐NMOSD (*n* = 33). These patients were classified based on the presence of comorbidities, either having one or ≥ 2, that is multiple comorbidities. Further classifications were made based on the presence of AID and CVC/RF (Figure 1).

In the AQP4‐NMOSD group 43.5% (*n* = 161) had at least one comorbidity. Within the comorbidity group, there were 40.4% (65) with AID and 34.8% (56) with CVC/RF. In the MOGAD cohort, 40.8% (*n* = 20) of patients had at least one comorbidity. Of those, 20.4% (4) were AID and 50% (10) were CVC/RF. In the DN‐NMOSD group, 36.4% (*n* = 12) of patients had at least one comorbidity, of which 33.3% (4) had CVC/RF and none had AID.

People with AQP4‐NMOSD (50%, *n* = 81/161) more frequently had multiple comorbidities compared to people with MOGAD (25%, *n* = 5/20) (*p* = 0.03), independent of age. Furthermore, people with AQP4‐NMOSD more frequently had AID (40.4%, *n* = 65/161) compared to MOGAD (20%, *n* = 4/20) (*p* = 0.09) and DN‐NMOSD (0% *n* = 0/12) (0.004), independent of age.

In AQP4‐NMOSD, the two most common CVC/RF comorbidities included hypertension (*n* = 29) and diabetes mellitus (*n* = 12), followed by dyslipidemia (*n* = 11). For MOGAD, hypertension (*n* = 6) was most prevalent, with fewer cases of diabetes mellitus (*n* = 2) and obesity (*n* = 3). In DN‐NMOSD, diabetes mellitus (*n* = 2) and hypertension (*n* = 1) were recorded. Autoimmune diseases were also frequent in AQP4‐NMOSD, including systemic lupus erythematosus (SLE) (*n* = 17), Sjögren's syndrome (*n* = 11), myasthenia gravis (*n* = 9), and autoimmune thyroiditis (Hashimoto's disease) (*n* = 5) (Table S1).

### 3.2 Demographics and Clinical Characteristics

#### 3.2.1 Sex and Age

The details of the demographic and clinical characteristics are provided in Table 1. AQP4‐NMOSD had a high female predominance in all comorbidity categories, ranging from 85.7% to 92.3% female. 55.2% of MOGAD patients without comorbidities were female, and 65% of those had at least one comorbidity. For DN‐NMOSD, patients without comorbidities consisted of 71.4% female, rising to 91.7% female in those with comorbidities (Table 1).

**TABLE 1:** Demographic and clinical characteristics across comorbidity status, autoimmune and cardiovascular comorbidity and risk factor status in AQP4‐NMOSD, MOGAD, and DN‐NMOSD patients.

	No comorbidity	At least one comorbidity	At least one autoimmune comorbidity	At least one cardiovascular comorbidities & risk factors
Number of patients				
AQP4‐NMOSD (*N* = 360)	199 (53.8%)	161 (43.5%)	65 (40.4%)	56 (34.8%)
MOGAD (*N* = 49)	29 (59.2%)	20 (40.8%)	4 (20%)	10 (50%)
DN‐NMOSD (*N* = 33)	21 (63.6%)	12 (36.4%)	0 (0%)	4 (33.3%)
Age (years), mean ± SD				
AQP4‐NMOSD	41.0 ± 13.5	49.8 ± 14.8*	50.10 ± 14*	52.9 ± 13.6*
MOGAD	31.6 ± 11.6	48.0 ± 14.8*	46.50 ± 10.4*	50.9 ± 14.4*
DN‐NMOSD	30.2 ± 7.85	41.7 ± 17.3*	—	58.2 ± 9.74*
*p*	< 0.001*	0.19	0.61	0.66
Sex, female, *N* (%)				
AQP4‐NMOSD	178 (89.4)	144 (89.4)	60 (92.3)	48 (85.71)
MOGAD	16 (55.2)	13 (65)	3 (75)	6 (60)
DN‐NMOSD	15 (71.4)	11 (91.7)	0 (0) *	4 (100)
*p*	< 0.001*	0.007*	0.007*	0.08
EDSS, median (IQR)				
AQP4‐NMOSD	3.5 (2–5.5)	3.5 (2–4.5)	3 (2–4.5)	3.5 (2.5–6)
MOGAD	2 (1–2)	3 (2–3.5) *	1.25 (0.6–1.9)	3 (1.3–3)
DN‐NMOSD	2 (1–2)	2.5 (1–3.5)	—	5 (5–5) *
*p*	< 0.001*	0.07	0.11	0.07
Age at onset (years), mean ± SD				
AQP4‐NMOSD	33.5 ± 13.7	42.7 ± 15.7*	42.7 ± 15.1*	45.6 ± 15.1*
MOGAD	28.6 ± 12.1	42.3 ± 17.2*	46.5 ± 10.4*	44.4 ± 17*
DN‐NMOSD	24.5 ± 8.27	35.9 ± 16.5*	—	47.8 ± 15.5*
*p*	0.003*	0.36	0.1	0.93
Time since onset (years), median (IQR)				
AQP4‐NMOSD	6.4 (2–11.7)	4.7 (2–10.1)	4.4 (1.9–11.3)	5.2 (1.9–9.9)
MOGAD	1.9 (0.7–3.6)	2.1 (0.1–7)	0.2 (0.1–0.3)	1.8 (1.1–5.1)
DN‐NMOSD	5.1 (2.3–8.8)	2.8 (1–10.8)	—	11.9 (8–14.3)
*p*	< 0.001*	0.64	0.07	0.73

*Note:* Statistics: *t*‐test/ANOVA for comparing two means/more than two means, with post hoc tests if needed; chi‐squared for proportion comparison, or Fisher's exact test when expected counts are low. The asterisk in each cell indicates a comparison of that cell to the no comorbidity group: *if *p* < 0.05. If there is no symbol, the comparison is not statistically significant.

Abbreviations: μm, micrometer; AQP4‐NMOSD, aquaporin‐4 positive neuromyelitis optica spectrum disorder; DN‐NMOSD, double negative neuromyelitis optica spectrum disorder; EDSS, Expanded Disability Status Scale; IQR, interquartile range; logMAR, logarithm of the minimum angle of resolution, lower logMAR values indicate better visual acuity; MOGAD, myelin oligodendrocyte glycoprotein antibody‐associated disease; *N*, number; ON, optic neuritis; SD, standard deviation.

The mean age was higher in the AQP4‐NMOSD group with at least one comorbidity (49.8 ± 14.8 years) (*p* ≤ 0.001), those with AID (50.1 ± 14 years) (*p* ≤ 0.001), and those with CVC/RF (52.9 ± 13.6 years) (*p* ≤ 0.001) compared to those with no comorbidity (41.0 ± 13.5 years). The mean age was higher in patients with comorbidities compared to those without in both MOGAD and DN‐NMOSD groups (Table 1).

Age at onset was higher in patients with comorbidities across all groups. The mean age at onset was higher in patients with at least one comorbidity (42.7 ± 15.7 years) (*p* < 0.001), those with AID (42.7 ± 15.1 years) (*p* ≤ 0.001), and those with CVC/RF (45.6 ± 15.1 years) (*p* ≤ 0.001) compared to those with no comorbidity (33.5 ± 13.7 years). The mean age at onset was higher in patients with comorbidities compared to those without in both MOGAD and DN‐NMOSD groups (Table 1).

**FIGURE 2:**
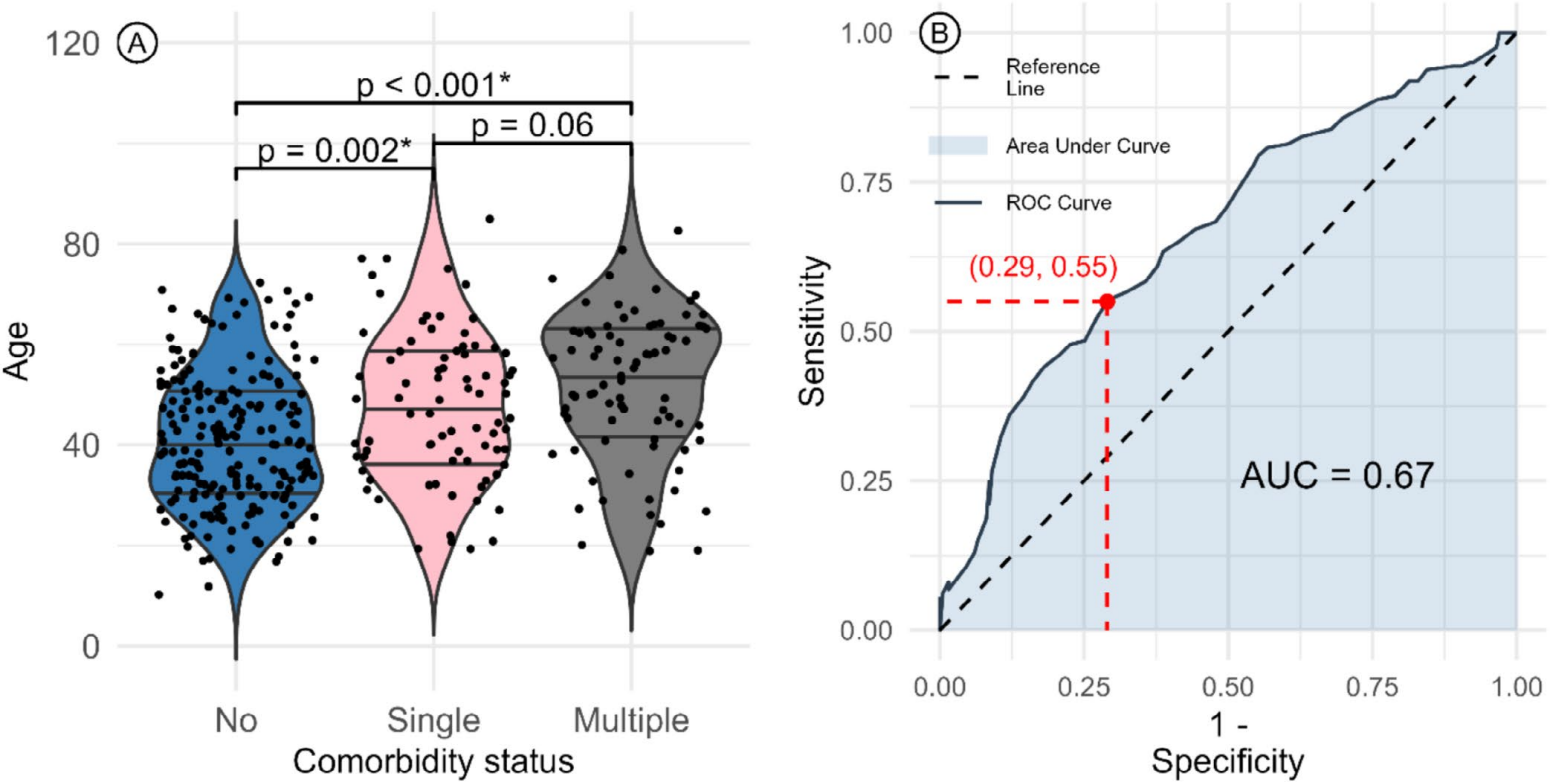
(A) Violin plot of age distribution by comorbidity in AQP4‐NMOSD. No: No comorbidity, Single: Single Comorbidity, Multiple: Multiple comorbidities. *Statistical significance is indicated where *p* < 0.05. (B) ROC Curve depicting the logistic regression model's performance in comorbidity prediction by age for AQP4‐NMOSD, with an AUC of 0.67. The optimal age cutoff is approximately 48.5 years, maximizing the sum of sensitivity and specificity at the given points of 0.55 and 0.71, respectively.

The age distribution of AQP4‐NMOSD patients, stratified by comorbidity status, is shown in Figure 2A. Patients with a single comorbidity (47.5 ± 20.5 years) were on average 7.5 years older than those without comorbidities (40.0 ± 19.5 years) (*p* = 0.002), while patients with multiple comorbidities (54.0 ± 19 years) were 14 years older than those without comorbidities (*p* < 0.001). However, in the comorbidity group in all three diseases, the age distribution did not show any significant differences: AQP4‐NMOSD 49.8 ± 14.8, MOGAD 48.0 ± 14.8, DS‐NMOSD 41.7 ± 17.3 (*p* = 0.19) (Table 1). To determine the threshold age for predicting comorbidity presence in the AQP4‐NMOSD subgroup, logistic regression analysis was performed. The aim was to ascertain the efficacy of patient age as a predictor for the development of comorbid conditions. The dataset was divided into two groups: those without comorbidities (*n* = 199) and those with at least one comorbid condition (*n* = 161). Receiver Operating Characteristic (ROC) analysis was conducted, which resulted in an Area Under the Curve (AUC) of 0.67. This AUC value reflects the logistic regression model's performance in comorbidity prediction as a function of age and indicates a moderate discriminative ability of the model to differentiate between patients with and without comorbidity. Furthermore, the analysis for the cutoff threshold gets maximized at a sensitivity of 0.55 and specificity of 0.71, which corresponds to the age threshold of 48.5 years, suggesting an increased likelihood of having a comorbidity probability beyond this age (Figure 2B).

#### 3.2.2 Disease‐Related Disability Characteristics

AQP4‐NMOSD patients with comorbidities had a similar median EDSS of 3.5 (IQR: 2–4.5) compared to 3.5 (IQR: 2–5.5) in those without. MOGAD patients with comorbidities showed a higher median EDSS of 3 (IQR: 2–3.5) versus 2 (IQR: 1–2) in those without (*p* = 0.006). DN‐NMOSD patients with CVC/RF comorbidities had a higher median EDSS of 5 (IQR: 5–5) compared to 2 (IQR: 1–2) in those without (*p* = 0.008).

#### 3.2.3 Association Between Presence of Comorbidities on Visual Outcome and OCT Parameters in AQP4‐NMOSD

Due to low sample sizes in the MOGAD and DN groups, the analysis on visual outcome and OCT parameters was only conducted in the AQP4‐NMOSD group. There were no differences in high contrast visual acuities between patients without comorbidities (mean = 0.1, range (0.1–0.33)) compared to those with comorbidities (mean = 0, range (0–0.1), *p* = 0.48). For the OCT analysis in AQP4‐NMOSD patients, the two mentioned models were performed. The comparison between the comorbidity groups for rejected and accepted scans using OSCAR‐IB criteria was performed [35, 36]. In the criteria, R stands for retinopathy, so scans with visible pathology influencing OCT measurements other than ON were excluded. The results showed that comorbidities did not affect the frequency of rejected scans, and the details are provided in Table S2.

Two models were applied. The first model analyzed eyes with and without ON separately, using random effects for inter‐eye variations and fixed effects for comorbidity, AID, and CVC/RF. The second, more complex model included these same effects but also added interactions between ON status and comorbidity, AID, or CVC/RF.

**FIGURE 3:**
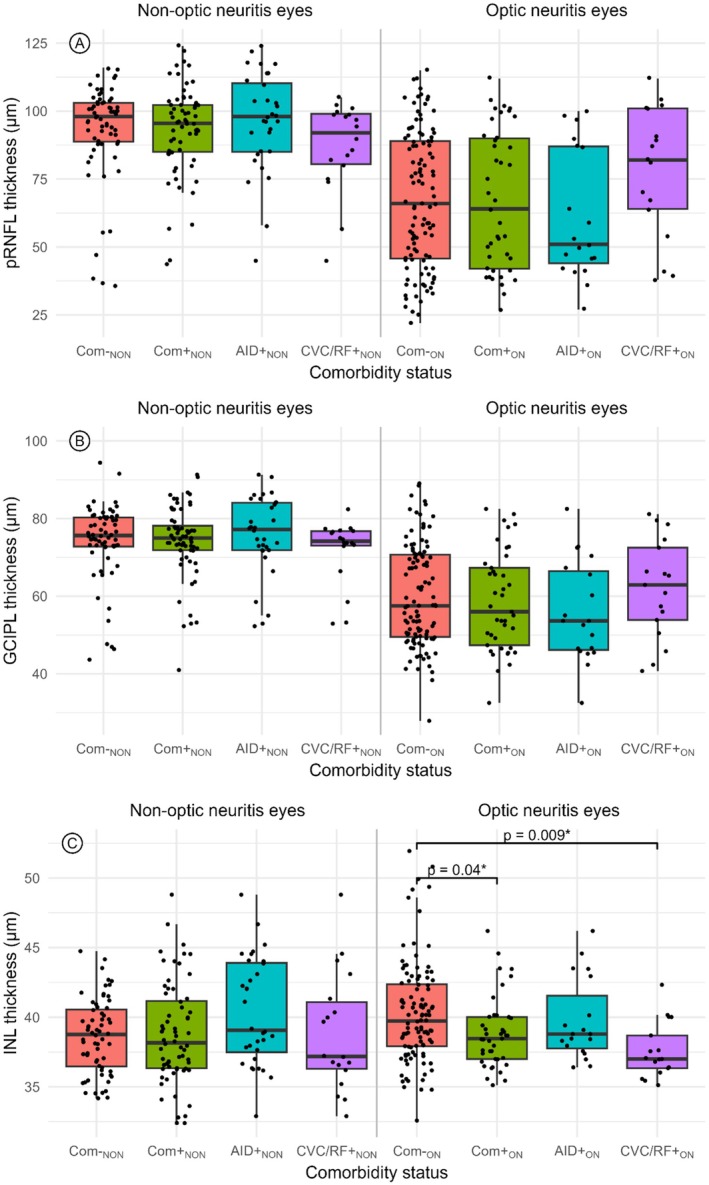
Boxplot of OCT parameters in the AQP‐NMOSD group. (A) Peripapillary Retinal nerve fiber layer (pRNFL) thickness, (B) ganglion cell‐inner plexiform layer (GCIPL) thickness, and (C) inner nuclear layer (INL) thickness comparing patients with and without a history of optic neuritis (ON). Number of ON eyes: No comorbidity *n* = 108; at least one comorbidity: *N* = 43; at least one autoimmune comorbidity: *N* = 21; at least one cardiovascular comorbidities & risk factors: *N* = 19. Statistics: Linear mixed‐effects modeling was applied separately to two subsets of eyes—those with ON and those without. This analysis included random effects to account for inter‐eye within‐subject variations and fixed effects for comorbidity, AID, and CVC/RF separately. *Statistical significance is indicated where *p* < 0.05. Abbreviations: Com−_NON_: Patients without comorbidities and without a history of ON; Com+_NON_: Patients with comorbidities but without a history of ON; AID+_NON_: Patients with autoimmune comorbidities but without a history of ON; CVC/RF+_NON_: Patients with cardiovascular comorbidities & risk factors but without a history of ON; Com−_ON_: Patients without comorbidities and without a history of ON; Com+_ON_: Patients with comorbidities and with a history of ON; AID+_ON_: Patients with autoimmune comorbidities and with a history of ON; CVC/RF+_ON_: Patients with cardiovascular comorbidities & risk factors and with a history of ON. Statistics: Linear mixed‐effects modeling was applied separately to two subsets of eyes—those with ON and those without. This analysis included random effects to account for inter‐eye within‐subject variations and fixed effects for comorbidity, AID, and CVC/RF separately. *Statistical significance is indicated where *p* < 0.05.

In the first model, both the pRNFL and the GCIPL showed no significant difference in thickness between individuals with comorbid conditions (*n* = 41) and those without (*n* = 108) (pRNFL: *B* = −0.91, SE = 5.44, *p* = 0.87; GCIPL: *B* = −1.47, SE = 2.82, *p* = 0.6). Additionally, no significant differences were observed in relation to AID or CVC/RF in these retinal layer thickness measurements. In this model, in eyes without ON, there was no significant difference in all comparisons (Figure 3).

However, in eyes with ON, the INL showed a significant reduction in thickness in people with comorbid conditions (*n* = 41) compared to the non‐comorbidity group (*n* = 108) (*B* = −1.52, SE = 0.41, *p* = 0.047). Eyes from people with CVC/RF (*n* = 17) had a significantly thinner INL thickness (mean = 37.6, SD = 2.0) compared to those without comorbidities (*n* = 108) (mean = 40.27, SD = 3.6), with a difference of −2.6 (*B* = −2.96, SE = 1.12, *p* = 0.009), independent of age.

In order to explain these findings, we performed further analyses between comorbidity types in the ON group. The annual ON relapse rate was lower in the comorbidity group (0.34 ± 0.50) than in the no comorbidity group (1.10 ± 5.49, *p* = 0.01). Within the comorbidity group, the annual ON relapse rate per person was higher in those with solely CVC/RF comorbidity (*n* = 42, 1.06 ± 3.33) compared to those with solely AID comorbidity (*n* = 48, 0.49 ± 0.98, *p* < 0.001).

Furthermore, in the second model, only ON showed a significant effect on the thickness of all three retinal layers. Specifically, for pRNFL thickness, ON was associated with a reduction (*B* = −25.22, SE = 4.18, *p* < 0.001). Similarly, for the GCIPL thickness, ON led to a significant decrease (*B* = −13.72, SE = 2.22, *p* < 0.001), and for INL thickness, ON had a positive effect (*B* = 1.61, SE = 0.67, *p* = 0.02). However, in this model, comorbidity status (including general comorbidity, AID, and CVC/RF), as well as the interaction between ON and these comorbidities, did not significantly impact the thickness of the retinal layers (Table 2).

**TABLE 2:** Impact of comorbidity status, autoimmune and cardiovascular comorbidity, and risk factor status on OCT parameters in AQP4‐NMOSD patients.

	*B*	SE	*p*	*R* ^2^_marg_‐*R* ^2^_cond_
Comorbidity				
pRNFL thickness				
Optic neuritis	−25.22	4.18	< 0.001*	*R* ^2^_marg_: 0.25 *R* ^2^_cond_: 0.63
Comorbidity	0.59	4.65	0.90	
Interaction of ON and comorbidity	−1.43	6.58	0.83	
GCIPL thickness				
Optic neuritis	−13.72	2.22	< 0.001*	*R* ^2^_marg_: 0.27 *R* ^2^_cond_: 0.66
Comorbidity	0.19	2.47	0.94	
Interaction of ON and comorbidity	−1.67	3.50	0.63	
INL thickness				
Optic neuritis	1.61	0.67	0.02*	*R* ^2^_marg_:0.05 *R* ^2^_cond_:0.75
Comorbidity	−0.06	0.74	0.93	
Interaction of ON and comorbidity	−1.46	1.04	0.16	
Autoimmune comorbidity				
pRNFL thickness				
Optic neuritis	−25.22	4.23	< 0.001*	*R* ^2^_marg_: 0.28 *R* ^2^_cond_: 0.64
AD	4.08	5.78	0.48	
Interaction of ON and autoimmune comorbidity	−10.14	8.65	0.24	
GCIPL thickness				
Optic neuritis	−13.72	2.30	< 0.001*	*R* ^2^_marg_: 0.28 *R* ^2^_cond_: 0.67
AD	1.69	3.14	0.59	
Interaction of ON and AD	−5.25	4.69	0.27	
INL thickness				
Optic neuritis	1.61	0.69	0.02	*R* ^2^_marg_: 0.04 *R* ^2^_cond_: 0.72
AD	1.39	0.93	0.14	
Interaction of ON and AD	−1.88	1.39	0.18	
Cardiovascular comorbidity and risk factor				
pRNFL thickness				
Optic neuritis	−24.87	4.37	< 0.001*	*R* ^2^_marg_: 0.21 *R* ^2^_cond_: 0.64
CVC/RF	−2.84	6.90	0.68	
Interaction of ON and CVD/RF	14.27	9.71	0.14	
GCIPL thickness				
Optic neuritis	−13.60	2.36	< 0.001*	*R* ^2^_marg_: 0.22 *R* ^2^_cond_: 0.69
CVC/RF	−1.04	3.71	0.78	
Interaction of ON and CVD/RF	3.96	5.22	0.45	
INL thickness				
Optic neuritis	1.62	0.69	0.02*	*R* ^2^_marg_: 0.08 *R* ^2^_cond_: 0.79
CVC/RF	−0.27	1.09	0.80	
Interaction of ON and CVD/RF	−2.69	1.52	0.08	

Abbreviations: AD, autoimmune disorders; AQP4‐NMOSD, aquaporin‐4 positive neuromyelitis optica spectrum disorder; B, estimate; CVC/RF, cardiovascular disorders and risk factors; GCIPL, ganglion cell‐inner plexiform layer; INL, inner nuclear layer; ON, optic neuritis; pRNFL, peripapillary retinal nerve fiber layer; *R* ^2^ conditional (*R* ^2^ cond), proportion of variance explained by both the fixed and random factors; *R* ^2^ marginal (*R* ^2^ marg), proportion of variance explained by the fixed factors alone; SD, standard deviation; SE, standard errors.

* Statistical significance is indicated where *p* < 0.05. The linear mixed‐effects modeling analysis included random effects to account for within‐subject inter‐eye variations, as well as fixed effects for optic neuritis, comorbidity status, and their interaction.

## 4 Discussion

In this study originating from the CROCTINO cohort [12, 34, 38], we aimed to determine the frequency and type of comorbidities associated with demographic and clinical features in AQP4‐NMOSD, MOGAD, and DSDN‐NMOSD. We further explored whether visual outcomes and retinal structural integrity are associated with the presence of comorbidities. The main finding was that people with AQP4‐NMOSD were more likely to have multiple comorbidities compared to MOGAD. Importantly, comorbidities in AQP4‐NMOSD were more likely to be of autoimmune origin compared to MOGAD or DN‐NMOSD. These relationships were independent of age. In patients with MOGAD or DN‐NMOSD and comorbidities, cardiovascular comorbidities and related risk factors were associated with more severe disability (higher EDSS) and in patients with AQP4‐NMOSD, with a higher annual ON relapse rate. This finding suggests that comorbidities have a negative impact on clinical outcomes. Moreover, in ON‐affected eyes, patients having AQP4‐NMOSD and comorbidities demonstrated a significant reduction in the thickness of the INL, indicating that changes in this layer may be associated with disease severity and prognosis in the presence of comorbidities. A proactive management of comorbidities should be considered in clinical practice.

AQP4‐NMOSD occurs in females with a disproportionate frequency of up to 9:1 as compared to males [39]. The current data indicate that the comorbidities group in all three diseases exhibited a high female predominance, with the highest female proportion observed in autoimmune comorbidities in AQP4‐NMOSD (92.3%). This pattern of results is consistent with the idea that autoimmune disease prevalence is greater in females due to specific factors. Among mechanisms proposed to contribute to the female propensity for most autoimmune diseases include endocrine/hormonal or genetic [40]/human leukocyte antigen (HLA) mechanisms [41]. However, while our sample showed a female predominance, this difference was not statistically significant, and the low number of males precluded a subgroup analysis. Therefore, we could not determine whether gender clustering of comorbidities contributes to differences in disease prognosis.

Interestingly, the mean age and age at onset were higher in the comorbidity group than in patients lacking comorbidity in all three conditions. The age distribution in the comorbidity group in all three diseases did not show any significant differences (Table 1). The age distribution in the comorbidity group in all three diseases did not show any significant differences (Table 1).

Approximately half of the AQP4‐NMOSD group experienced at least one comorbidity, suggesting that older people are more likely to develop comorbid conditions. This underscores the importance of monitoring for comorbidities in older people, as these conditions potentially could lead to broader health implications.

Limited studies of comorbidities reported increased frequency of comorbid conditions in AQP4‐NMOSD in line with our findings [42, 43, 44, 45, 46]. However, our study included a larger number of patients and compared AQP4‐NMOSD with MOGAD and DN‐NMOSD, groups noting different pattern characteristics of comorbidities in these disease entities [29]. Of note, CVC/RF did not appear to be different across the three disease entities highlighting the potential gain in screening of CVC/RF strategies. Interestingly, the annual relapse rate in AQP4‐NMOSD was significantly higher with CVC/RF comorbidities compared to AQP4‐NMOSD with only autoimmune comorbidities. It may be speculated that this is a general phenomenon, that the coexistence of two or more AID may ameliorate the disease course.

Similarly, MS studies suggest that the most common comorbidities in MS are CVC/RF including obesity, hypertension, hyperlipidemia, and type 2 diabetes mellitus (T2DM) [47]. Furthermore, several studies indicate that comorbidities in MS are associated with disability progression, lesion accrual on CNS MRI, lower quality of life, hospitalizations, and mortality [48, 49, 50].

In the current study, we observed that the INL thicknesses in ON eyes were lower in the AQP4‐NMOSD group with comorbidities, and with a more pronounced reduction in people with CVC/RF comorbidity compared to the no comorbidity group. The INL contains both neuronal and glial cells, including AQP4‐expressing Müller cells [51]. Of note, the retinal vasculature of the deep capillary network is located in the INL [52].

In MS, overlapping processes of INL thickening during inflammatory disease stages and INL thinning during the more progressive disease stage have been described [53]. An OCT study showed faster thinning of INL and the outer plexiform layer in progressive MS compared to the age‐matched RRMS group and healthy controls [54]. Moreover, a recent study demonstrated retinal degeneration, as the INL thickness decreased in the earlier phases of primary progressive MS and late phases of SPMS [55], in line with pathology findings in post‐mortem MS eyes [53]. One challenge with these studies is that the timing from ON to OCT assessment is not known and therefore it is not possible to separate the resolution of inflammation related INL edema (also called pseudoatrophy) from true atrophy. In another study in MS, the cumulative effect of comorbidities significantly influenced pRNFL and the GCIPL [56]. However, in MS, the impact of comorbidities on INL has not yet been investigated. In AQP4‐NMOSD, we previously reported that INL was thicker in the affected‐ON eye compared with HC [38]. However, in the first model of analysis of the present study in AQP4‐NMOSD with comorbidities, a reduction in INL thickness was observed, which may suggest a localized neurodegenerative process at this site, insufficient immunological repair mechanisms, or alternatively another non–disease‐specific process due to the presence of comorbidity. We did not find significant differences in the thickness of the pRNFL and the GCIPL, suggesting that these layers may not be as sensitive to changes associated with comorbid conditions in this group. However, the second complex model showed that ON status, but neither comorbidity status (including general comorbidity, AID, and CVC/RF) nor the interaction between ON and these comorbidities impacted retinal layer thickness. INL swelling is an inflammatory process, and its resolution is highly dynamic, [53] so our results may primarily reflect ON‐related thickness changes. Consequently, these results should be viewed as exploratory, and they warrant further validation in larger, prospectively designed cohorts.

This finding highlights the importance of further investigation into how systemic conditions influence neuroinflammatory and neurodegenerative processes in the retina. One important strength of our study was that patients originated from a large cohort diagnosed with AQP4‐NMOSD, MOGAD, and DN‐NMOSD from multiple centers worldwide, which increases the generalizability of the findings. A limitation was the cross‐sectional design of our study instead of a longitudinal design for the purpose; however, our cohort represented all disease stages. The retrospective design of the CROCTINO dataset might have led to inaccuracies in the documentation of comorbidities, particularly regarding onset, duration, treatment profile, and their impact on disease outcomes. Specific risk factors, such as smoking, were not systematically collected. The absence of standardized methods for collecting and categorizing comorbidity data might have led to inconsistencies in the recording of onset and duration of comorbidity. Thus, certain comorbidities, such as diabetes or orthopedic conditions, could independently influence OCT or EDSS findings, respectively, regardless of AQP4‐NMOSD/MOGAD disease activity. Similarly, patients with autoimmune comorbidities may have received more intensive immunosuppressive treatment, potentially affecting disease outcomes. The relatively small sample size within comorbidity subgroups further limited statistical power. Future directions should focus on prospective studies with standardized data collection to ensure accuracy and consistency in reporting comorbid conditions.

Further research is needed to explore the mechanisms through which systemic diseases affect retinal structures and to enhance the sensitivity of OCT technology in detecting subtle changes to validate these observations would be an obvious continuation. Comorbidities may influence NMOSD, MOGAD, and DSDN‐NMOSD‐related treatment effectiveness, safety, tolerability, and adherence. Therefore, knowledge of comorbid conditions is critical. Refining comorbidity management strategies will be crucial to better address the overall health and vision preservation needs of these patients.

## 5 Conclusion

The CROCTINO dataset revealed a higher prevalence of multiple comorbidities and autoimmune disorders in AQP4‐NMOSD than in MOGAD and DN‐NMOSD. Cardiovascular comorbidities and related risk factors were common across all groups and correlated with worse clinical outcomes, particularly in MOGAD and DN‐NMOSD. In AQP4‐NMOSD, cardiovascular comorbidities and related risk factors led to a higher ON relapse rate than in AQP4‐NMOSD with autoimmune comorbidities. Our results indicate a reduction in INL thickness in the AQP4‐NMOSD comorbidity group. Future prospective research should include a focus on how systemic diseases and effective management of comorbidities affect retinal structures in these disease groups.

## Author Contributions

**Sara Samadzadeh:** conceptualization, investigation, funding acquisition, writing – original draft, methodology, validation, visualization, writing – review and editing, software, formal analysis, project administration, data curation, supervision, resources. **Frederike Cosima Oertel:** writing – review and editing, methodology, resources, validation. **Hadi Salih:** writing – review and editing. **Ting‐Yi Lin:** writing – review and editing, methodology. **Seyedamirhosein Motamedi:** writing – review and editing, resources. **Claudia Chien:** writing – review and editing, resources. **Lawrence J. Cook:** writing – review and editing, resources. **Marco Aurélio Lana‐Peixoto:** writing – review and editing, resources. **Mariana Andrade Fontenelle:** writing – review and editing, resources. **Ho Jin Kim:** writing – review and editing, resources. **Jae‐Won Hyun:** writing – review and editing, resources. **Su‐Kyung Jung:** writing – review and editing, resources. **Jaqueline Palace:** writing – review and editing, resources. **Adriana Roca‐Fernandez:** writing – review and editing, resources. **Maria Isabel Leite:** writing – review and editing, resources. **Srilakshmi M. Sharma:** writing – review and editing, resources. **Fereshteh Ashtari:** writing – review and editing, resources. **Rahele Kafieh:** resources, writing – review and editing. **Alireza Dehghani:** writing – review and editing, resources. **Mohsen Pourazizi:** resources, writing – review and editing. **Lekha Pandit:** writing – review and editing, resources. **Anitha Dcunha:** resources, writing – review and editing. **Orhan Aktas:** writing – review and editing, resources. **Marius Ringelstein:** resources, writing – review and editing. **Philipp Albrecht:** writing – review and editing, resources. **Eugene F. May:** writing – review and editing, resources. **Caryl Tongco:** writing – review and editing, resources. **Letizia Leocani:** resources, writing – review and editing. **Marco Pisa:** writing – review and editing, resources. **Marta Radaelli:** writing – review and editing, resources. **Bernardo Sánchez‐Dalmau:** writing – review and editing, resources. **Elena H. Martinez‐Lapiscina:** writing – review and editing, resources. **Hadas Stiebel‐Kalish:** resources, writing – review and editing. **Mark A. Hellmann:** writing – review and editing, resources. **Itay Lotan:** resources, writing – review and editing. **Sasitorn Siritho:** writing – review and editing, resources. **Jérôme de Seze:** resources, writing – review and editing. **Thomas Senger:** writing – review and editing, resources. **Joachim Havla:** resources, writing – review and editing. **Romain Marignier:** writing – review and editing, resources. **Caroline Froment Tilikete:** resources, writing – review and editing. **Alvaro Cobo‐Calvo:** writing – review and editing, resources. **Denis Bichuetti:** resources, writing – review and editing. **Ivan Maynart Tavares:** writing – review and editing, resources. **Kerstin Soelberg:** resources, writing – review and editing. **Ayse Altintas:** writing – review and editing, resources. **Rengin Yildirim:** resources, writing – review and editing. **Uygur Tanriverdi:** writing – review and editing, resources. **Anu Jacob:** resources, writing – review and editing. **Saif Huda:** writing – review and editing, resources. **Zoe Rimler:** resources, writing – review and editing. **Allyson Reid:** writing – review and editing, resources. **Yang Mao‐Draayer:** resources, writing – review and editing. **Pablo Villoslada:** writing – review and editing, resources. **Ibis Soto de Castillo:** resources, writing – review and editing. **Ari Green:** writing – review and editing, resources. **Axel Petzold:** resources, writing – review and editing. **Michael R. Yeaman:** writing – review and editing, resources. **Terry J. Smith:** resources, writing – review and editing. **Alexander U. Brandt:** writing – review and editing, resources. **Hanna G. Zimmermann:** writing – review and editing, resources, conceptualization, investigation, funding acquisition, writing – original draft, methodology, validation, formal analysis, project administration, supervision, software, visualization, data curation. **Friedemann Paul:** conceptualization, investigation, funding acquisition, writing – original draft, methodology, validation, writing – review and editing, project administration, supervision, resources. **Nasrin Asgari:** conceptualization, investigation, funding acquisition, writing – original draft, methodology, validation, visualization, writing – review and editing, software, formal analysis, project administration, resources, supervision, data curation.

## Conflicts of Interest

F.C.O. currently receives research support from the Hertie foundation, the Deutsche Forschungsgemeinschaft (DFG) and Novartis, all unrelated to this study. She received fellowship support from the American Academy of Neurology, National MS Society until 2023. She is a member of the working committee of the International Multiple Sclerosis Visual System (IMSVISUAL) Consortium. H.G.Z. reports grants from Novartis, unrelated to this study H.J.K. has received a grant from the National Research Foundation of Korea and research support from AprilBio, Eisai, and UCB; received consultancy/speaker fees from Alexion, Altos Biologics, AstraZeneca, Biogen, Daewoong Pharmaceutical, Eisai, GC Pharma, Handok Pharmaceutical, Kaigene, Kolon Life Science, MDimune, Merck Serono, Mitsubishi Tanabe Pharma, Roche, and Sanofi Genzyme; is a co‐editor for the Multiple Sclerosis Journal; and an associated editor for the *Journal of Clinical Neurology*. J.P. has received support for scientific meetings and honorariums for advisory work from Merck Serono, Novartis, Chugai, Alexion, Roche, Medimmune, Amgen, Vitaccess, UCB, Mitsubishi, Amplo, and Janssen, grants from Alexion, Argenx, Clene, Roche, Medimmune, and Amplo biotechnology, patent ref P37347WO and license agreement Numares multimarker MS diagnostics, and shares in AstraZeneca. Her group has been awarded an ECTRIMS fellowship and a Sumaira Foundation grant to start later this year. A Charcot fellow worked in Oxford 2019–2021. She acknowledges partial funding to the trust by National Health Service (NHS) Highly Specialised Services England. She is on the medical advisory boards of the Sumaira Foundation and MOG project charities, is a member of the Guthy Jackson Foundation Charity, and is on the Board of the European Charcot Foundation and the steering committee of MAGNIMS and the UK NHSE IVIG Committee, and chairman of the NHSE neuroimmunology patient pathway, and ECTRIMS Council member on the educational committee since June 2023. On the ABN advisory groups for MS and neuroinflammation and neuromuscular diseases, A.R.‐F. is sponsored by Abide Therapeutic outside of the submitted work and reports no potential conflicts of interest. P.A. received, with approval of the Rector of Heinrich‐Heine University and the CEO of University of Düsseldorf Hospital, personal fees, research grants, and non‐financial support from Allergan, Biogen, Celgene, Janssen Cilag, Ipsen, Merck Serono, Merz Pharmaceuticals, Novartis, and Roche; personal fees and non‐financial support from Bayer Healthcare, Teva, and Sanofi‐Aventis/Genzyme; and grants from the German Research Foundation (DFG), all outside the submitted work. M.R. received speaker honoraria from Novartis, Bayer Vital GmbH, Roche, Alexion, and Ipsen and travel reimbursement from Bayer Schering, Biogen Idec, Merz, Genzyme, Teva, Roche, and Merck, none related to this study. H.S.‐K. has received support for scientific meetings from Roche. A.U.B. is co‐founder and shareholder of Motognosis GmbH and Nocturne GmbH. He is named as inventor on several patent applications regarding MS serum biomarkers, OCT image analysis, and perceptive visual computing. A.U.B. is now a full‐time employee and holds stocks of Eli Lilly and Corporation. His contribution to this work is his own and does not reflect Eli Lilly and Corporation. F.P. reports research grants and speaker honoraria from Bayer, Teva, Genzyme, Merck, Novartis, MedImmune, and is a member of the steering committee of the OCTIMS study (Novartis), all unrelated to this work. E.H.M.‐L. received funding from the Instituto de Salud Carlos III (Spain) and Fondo Europeo de Desarrollo Regional (FEDER—JR16/00006), Grant for MS Innovation, Fundació Privada Cellex, and Marató TV3 Charitable Foundation, and is a researcher in the OCTIMS study, an observational study (that involves no specific drugs) to validate SD‐OCT as a biomarker for MS, sponsored by Novartis, and has received honoraria and travel support for international and national meetings over the last 3 years from Biogen, Novartis, Roche, and Genzyme. She is a member of the working committee of the International Multiple Sclerosis Visual System (IMSVISUAL) Consortium. M.A.L.‐P. has received funding for travel and speaker honoraria from Novartis, Horizon Therapeutics, and Roche. M.I.L. reported being involved in aquaporin 4 testing, receiving salary from the NHS National Highly Specialised Commissioning Group for Neuromyelitis Optica, UK, being supported by the National Institute for Health Research Oxford Biomedical Research Centre, UK, and receiving speaking honoraria and travel grants from Biogen Idec, and a travel grant from Novartis. S.S.I. received funding for travel and speaker honoraria from Merck Serono (Thailand), Roche (Thailand), DKSH (Thailand), Pacific Healthcare (Thailand), Eisai (Thailand), Biogen Idec, UCB (Thailand), and Novartis. A.A. reports personal fees from received honoraria for giving educational presentations on multiple sclerosis, NMOSD, and neuroimmunology from Novartis and Alexion. Dr. Altintas has received travel and registration coverage for attending several national and international meetings from Merck‐Serono. A.J. has received compensation for advisory board, consulting, meeting attendance, and speaking from Biogen, Terumo‐BCT, Genentech, Shire, and Chugai Pharmaceuticals. S.H. is partly funded by NHS Highly Specialised Services England to run an NMO UK service. S.H. has received research funding from the NIHR. R.M. serves on the scientific advisory board for MedImmune and has received funding for travel and honoraria from Biogen, Merck Serono, Novartis, Sanofi‐Genzyme, Roche, and Teva. D.B. has received speaking/consulting honoraria from Bayer Health Care, Biogen Idec, Merck, Sanofi‐Genzyme, TEVA, and Roche, and had travel expenses to scientific meetings sponsored by Bayer Health Care, Merck Serono, TEVA, and Roche. J.H. reports a grant for OCT research from the Friedrich‐Baur‐Stiftung and Merck, personal fees and non‐financial support from Merck, Alexion, Novartis, Roche, Celgene, Biogen, Bayer, and Horizon, and non‐financial support from the Sumaira Foundation and Guthy‐Jackson Charitable Foundation, all outside the submitted work. L.L. received honoraria for consulting services from Merck, Roche, Biogen, and for speaking activities from Teva; research support from Merck, Biogen, Novartis; travel support from Merck, Roche, Biogen, and Almirall. O.A. has received honoraria for speaking/consultation and travel grants from Bayer Healthcare, Biogen Idec, Chugai, Novartis, Medimmune, Merck Serono, and Teva, and research grants from Bayer Healthcare, Biogen Idec, Novartis, and Teva. M.R. received speaker honoraria from Novartis, Bayer Vital GmbH, and Ipsen, and travel reimbursement from Bayer Schering, Biogen Idec, Merz, Genzyme, Teva, and Merck, none related to this study. P.V. has received consultancy fees and holds stocks in Bionure investments, Accure Therapeutics, Attune Neurosciences, Adhera Health, Clight, QMENTA, and NeuroPrex. J.‐W.H. has received a grant from the National Research Foundation of Korea. A.P. received salary support—grant to Biomedical Research Centre at Moorfields Eye Hospital and UCL Institute of Ophthalmology. Y.M.‐D. has served as a consultant and/or received grant support from: Acorda, Bayer Pharmaceutical, Biogen Idec, Celgene, EMD Serono, Genzyme, Novartis, Questor, Chugai, and Teva Neuroscience, and is currently supported by grants from NIH NIAID Autoimmune Center of Excellence: UM1‐AI110557; NIH NINDS R01‐NS 080821. M.R.Y. is founder of NovaDigm Therapeutics, Inc., ImmunoTx, LLC, Tegos Therapeutics, LLC, and Metacin, Inc. He is the principal inventor on United States and international patents regarding anti‐infectives, immunotherapeutics, and vaccines. He has received research funding from the National Institutes of Health (NIH), National Institute of Allergy & Infectious Diseases (NIAID) and Department of Defense (DOD), United States of America. He has received speaker or advisory honoraria from Alexion/AstraZeneca, Horizon/Amgen, and Genentech/Roche. He serves as Chair Advisor to the Guthy‐Jackson Charitable Foundation. J.S. has nothing to disclose. The remaining co‐authors have nothing to disclose.

## Supporting information


Appendix S1.

## Data Availability

The data that support the findings of this study are available from the corresponding author upon reasonable request.
