# Supplementary material for: Comorbidities Are Associated With Unfavorable Outcome in Aquaporin‐4 Antibody Positive Neuromyelitis Optica Spectrum Disorders and Myelin Oligodendrocyte Glycoprotein Antibody‐Associated Disease: Exploratory Study From the CROCTINO Cohort
Source: Eur J Neurol. 2025 Jun 9;32(6):e70214. doi: 10.1111/ene.70214 (PMC12146790; doi:10.1111/ene.70214)
Supplement: Supplementary file 1 — Appendix S1. [file ENE-32-e70214-s001.docx]

| **Supplementary table 1: Comorbid Conditions across AQP4-NMOSD, MOGAD, and DN Patients** | | | |
| --- | --- | --- | --- |
|  | **AQP4-Pos-NMOSD** | **MOGAD** | **DN** |
| **Main Categories** | **Conditions** | **Conditions** | **Conditions** |
| Cardiovascular Disorders and Risk Factors^#^ | Hypertension (29 Occurrences); Heart Failure; Premature ventricular contraction (RVOT origin); Mitral valve prolapse; Non-ST-elevation myocardial infarction Pulmonary Embolism (4 Occurrences); Deep Venous Thrombosis (5 Occurrences); Arm vein thrombosis; Raynaud's Phenomenon (2 Occurrences); Aortic Valve Replacement; Coronary Artery Bypass Graft (CABG); Pericardial Disease; Cerebrovascular Accident (Stroke); Coronary artery disease (3 Occurrences); Subarachnoid Hemorrhage; Protein S Deficiency; Peripheral Vascular Disease; Pre-eclampsia; Sclerotic Aortic Valve; Atrial Septal Defect; Carotid Aneurysm  Diabetes Mellitus (12 Occurrences); Type 1 Diabetes (2 Occurrences); Steroid-induced Diabetes (2 Occurrences); Mellitus Gestational Diabetes; Hypercholesterolemia (8 Occurrences); Dyslipidemia (3 Occurrences); Prediabetes; Obesity (2 Occurrences) | Hypertension (6 Occurrences) Supraventricular Tachycardia; Diabetes Mellitus (2 Occurrences); Obesity (3 Occurrences), Prediabetes, Nicotine Dependence | Hypertension; Supraventricular Tachycardia; Pulmonary Embolism; Diabetes Mellitus (2 Occurrences); Nicotine Dependence |
| Autoimmune Disorders^#^ | Systemic Lupus Erythematosus (17 Occurrences); Sjögren's Syndrome (11 Occurrences); Autoimmune Neutropenia (2 Occurrences); Mixed Connective Tissue Disease; Antiphospholipid Antibodies Syndrome (3 Occurrences); Limited Cutaneous Systemic Sclerosis (Crest Syndrome); Behçet's Syndrome; Myeloperoxidase (MPO) Positive Vasculitis; Not Categorized Autoimmune Disorder; Autoimmune Thyroiditis (Hashimoto's Thyroiditis) (5 Occurrences); Hyperthyroidism (Grave's Disease) (7 Occurrences); Coeliac Disease (2 Occurrences); Rheumatoid Arthritis; Immune Thrombocytopenic Purpura (ITP); Autoimmune Hemolytic Anemia; Myasthenia Gravis (9 Occurrences); Ocular Myasthenia Gravis; Type 1 Diabetes (2 Occurrences); Vitamin B12 Deficiency (2 Occurrences); Pernicious Anemia; Psoriasis (2 Occurrences); Bullous Pemphigoid; Sarcoidosis; Ulcerative Colitis; Primary Sclerosing Cholangitis; Raynaud's Phenomenon (2 Occurrences). | Antiphospholipid Syndrome; Psoriasis; Vitamin B12 Deficiency (3 Occurrences) |  |
| Endocrine Disorders | Autoimmune Thyroiditis (Hashimoto's Thyroiditis) (5 Occurrences); Hyperparathyroidism (2 Occurrences); Vitamin D Deficiency (2 Occurrences); Autonomic thyroid nodule; Diabetes Mellitus (12 Occurrences); Type 1 Diabetes (2 Occurrences); Steroid-induced Diabetes (2 Occurrences); Mellitus Gestational Diabetes; Hypercholesterolemia (8 Occurrences); Dyslipidemia (3 Occurrences); Goiter; Nodular goiter (2 Occurrences); Thyroid nodule; Disease of the thyroid gland (2 Occurrences); Hypothyroidism (17 Occurrences); Hyperthyroidism (Grave's disease) (7 Occurrences); Obesity (2 Occurrences); Parathyroid gland enlargement; Vitamin D Deficiency; Thyroid Hyperplasia; Prediabetes | Osteoporosis, Vitamin B12 Deficiency (3 Occurrences), Diabetes Mellitus (2 Occurrences); Obesity (3 Occurrences), Prediabetes, Thyroid Hyperplasia | Hypothyroidism (2 Occurrences); Diabetes Mellitus (2 Occurrences); Nicotine Dependence |
| Psychiatric Disorders | Psychiatric Disorder; Depressive Syndrome; Major Depressive Disorder (2 Occurrences); Bipolar Disorder; Depression and Anxiety Disorders (2 Occurrences); Attention-Deficit/Hyperactivity Disorder | Major Depressive Disorder; Anxiety and Panic Disorder; Nicotine Dependence | Major Depressive Disorder, Anxiety Disorder |
| Neurological Disorders | Mononeuropathy Multiplex; Cerebral Aneurysm; Sinus Vein Thrombosis; Restless Legs Syndrome; Migraine (6 Occurrences); Epileptic Seizures; Epilepsy (2 Occurrences); Tinnitus; Cerebral Palsy; Myasthenia Gravis (9 Occurrences); Neuropathy | Encephalitis | Sensorimotor Neuropathy with Demyelinating Features |
| Respiratory Disorders | Allergic Rhinitis; Tonsillitis; Non-Specific Interstitial Pneumonia; Asthma (4 Occurrences); Obstructive Sleep Apnea (3 Occurrences); Sarcoidosis | - | Asthma |
| Gastrointestinal Disorders | Chronic Hepatic Injury; Toxic Hepatitis; Chronic Hepatitis C (2 Occurrences); Hepatitis B (2 Occurrences); Hepatic Steatosis; Gastric Ulcer; Gastro-Esophageal Reflux Disease; Acute Appendicitis (3 Occurrences); Cholelithiasis (Gallstones); Acute Cholecystitis; Acute Or Chronic Pancreatitis; Coeliac Disease (2 Occurrences); Ulcerative Colitis; Parotitis; Dysplastic Colon; Primary Sclerosing Cholangitis; Non-Alcoholic Steatohepatitis; Hepatomegaly | Gastric Perforation; Irritable Bowel Syndrome (IBS) | - |
| Neoplastic Disorders | Multiple Basal Cell Carcinomas; Benign Tumor Of The Lacrimal Gland And Duct; Thyroid Carcinoma; Hemangioma Of The Left Hip; Colorectal Cancer; Colon Adenoma; Hodgkin's Disease; Breast Cancer (2 Occurrences); Cervical Carcinoma; Squamous Cell Carcinoma; Chronic Lymphocytic Lymphoma; Lymphoma; Cutaneous Lymphoma | - | Malignant Neoplasms of The Unspecified Site; Benign Breast Mass/Tumor |
| Rheumatologic Disorders | Systemic Lupus Erythematosus (17 Occurrences); Sjögren's Syndrome (11 Occurrences); Intervertebral Disc Protrusion (2 Occurrences); Mixed Connective Tissue Disease; Antiphospholipid Antibodies Syndrome (3 Occurrences); Limited Cutaneous Systemic Sclerosis (Crest Syndrome); Behçet's Syndrome; Myeloperoxidase (MPO) Positive Vasculitis; Raynaud's Phenomenon (2 Occurrences); Thoracic/ Lumbar Spinal Compression; Hyperkyphosis; Scoliosis; Spinal Stenosis (3 Occurrences); Bilateral Bouchard's Osteoarthritis/Knee Osteoarthritis (2 Occurrences); Osteoporosis With Multiple Fractures (7 Occurrences); Osteopenia (2 Occurrences); Arthralgia; Rheumatoid Arthritis; Inflammatory Arthritis; Calcific Tendonitis; Chronic Back Pain; Avascular Necrosis; Fibromyalgia (2 Occurrences); Bursitis; Adhesive Capsulitis (Reeves); Rhabdomyolysis | Antiphospholipid Syndrome; Spinal Stenosis; Polymyalgia Rheumatica | Scoliosis |
| Hematologic Disorders | Autoimmune Neutropenia (2 Occurrences); Thrombocytopenia; Iron-Deficiency Anemia/Anemia (5 Occurrences); Recurrent Leukopenia; Immune Thrombocytopenic Purpura (ITP); Autoimmune Hemolytic Anemia; Vitamin B12 Deficiency (2 Occurrences); Pernicious Anemia; Intermittent Lymphopenia/Leukopenia Leukopenia; Drug-Induced Lymphopenia | Iron Deficiency Anemia; Vitamin B12 Deficiency (3 Occurrences) | - |
| Dermatologic Disorders | Cutaneous Nodular Amyloidosis; Bullous Pemphigoid; Prurigo; Psoriasis (2 Occurrences); Pityriasis Versicolor; Skin Warts; Pityriasis Versicolor | Psoriasis | - |
| Genitourinary Disorders | Proteinuria; Chronic Kidney Disease (2 Occurrences); Recurrent Cystitis; Neurogenic Bladder Dysfunction; (Acute) Renal Failure (2 Occurrences); Kidney Stones (Renal Calculi) (2 Occurrences); Urinary Urgency/Urge Incontinence; Renal Anomaly; Genitourinary Disorders (2 Occurrences); Pregnancy Threatened Abortion; Recurrent Miscarriages, Premature Infant Death; Uterine Polyp; Polycystic Ovarian Syndrome (3 Occurrences); Adenomyosis; Myoma; Endometrial Hyperplasia; Premature Menopause | - | Chronic Kidney Disease Stage G4 |
| Infectious Diseases | Leprosy; Pneumocystis Pneumonia with Sepsis; Sepsis; Epiglottic Abscess; Tuberculosis (2 Occurrences); Herpes Zoster (Shingles) Due To Varicella-Zoster (2 Occurrences); Onychomycosis | - | Pneumocystis Jirovecii Pneumonia |
| Ophthalmic Disorders | Bilateral/Unilateral Cataract With/Without Lens Replacement (14 Occurrences); Bilateral/Unilateral Cataracta Incipiens (4 Occurrences); Macular Changes (3 Occurrences); Glaucoma (5 Occurrences); Diabetic Retinopathy; Ocular Deviation (Strabismus); Bilateral Optic Disc Elevation; Bilateral Filamentary Keratopathy; Vitreous Detachment Without Retinal Involvement; Cystoid Macular Edema (Irvine-Gass Syndrome); Diabetic Macular Edema; Bilateral Keratoconjunctivitis Sicca (Dry Eye Syndrome) (2 Occurrences); Ocular Myasthenia Gravis; Marginal Keratitis; Epiretinal Membrane (2 Occurrences); Ocular Toxocariasis; Cytomegalovirus (CMV) Retinitis; Retinal Arteriolar Constriction | Glaucoma; Cataract; Lens Replacement; Macular Changes; Ocular Deviation (Strabismus) | Cataract Conditions; Diabetic Macular Edema; Diabetic Retinopathy |
| *Injuries And Other External Causes | Spinal Or Head Injury/Disorder/Fracture (7 Occurrences); Broken Ankle; Hip Fracture; Distal Fibula Fracture; Congenital Cleft Palate | Spinal Or Head Injury; Lumbar Vertebrae Fractures | Spinal Or Head Injury; Below-Knee Amputation; Congenital Cleft Palate |
| *For completeness, we have included the category injuries and other external causes; however, it is not considered as a chronic condition in any of our analyses.  ^#^These two groups contain all relevant disease items from other groups as well. For example, diabetes mellitus is categorized both as a cardiovascular risk factor and an organ-based endocrine disorder and autoimmune thyroiditis is classified as both an endocrine and autoimmune disorder. | | | |

| **Supplementary table 2:** OCT Scan Acceptance After OSCAR-IB Criteria Check | | | |
| --- | --- | --- | --- |
| Scan Type | Comorbidity Status | Accepted | Rejected |
| Peripapillary | With at least one comorbidity | 203 | 21 |
|  | No comorbidity | 139 | 19 |
|  | Chi-Squared Test | X-squared = 0.44, df = 1, p-value = 0.5 | |
| Macula | With at least one comorbidity | 115 | 43 |
|  | No comorbidity | 181 | 43 |
|  | Chi-Squared Test | X-squared = 2.97, df = 1, p-value = 0.08 | |
| The table compares OCT scan acceptance rates between patients with and without comorbidities. All p-values > 0.05, indicating non-significance. OCT: Optical coherence tomography. | | | |
